# Tixagevimab-Cilgavimab Effectively Prevents COVID-19 Infection in Patients with End-Stage Kidney Disease

**DOI:** 10.3390/v17091216

**Published:** 2025-09-06

**Authors:** Noppakao Kongtal, Watchara Pichitsiri, Supinda Sirilak, Anyarin Wannakittirat, Busakorn Sontham, Sagoontee Inkate, Theerachai Thammathiwat

**Affiliations:** 1 Division of Nephrology, Department of Medicine, Faculty of Medicine, Naresuan University, Phitsanulok 65000, Thailand; 2 Excellence Center in Renal Care, Naresuan University Hospital, Faculty of Medicine, Naresuan University, Phitsanulok 65000, Thailand; 3 Renal Unit, Nursing Department, Naresuan University Hospital, Phitsanulok 65000, Thailand; 4 Department of Family Medicine, Faculty of Medicine, Naresuan University, Phitsanulok 65000, Thailand

**Keywords:** long-acting antibody, COVID-19, end-stage kidney disease, dialysis

## Abstract

Patients with end-stage kidney disease (ESKD) often exhibit suboptimal responses to COVID-19 vaccination. Tixagevimab-Cilgavimab, a neutralizing long-acting antibody (LAAB), has demonstrated effectiveness in preventing severe COVID-19 and hospitalization among immunocompromised populations. This study aimed to evaluate the efficacy and safety of Tixagevimab-Cilgavimab in ESKD patients receiving hemodialysis, peritoneal dialysis, or kidney transplantation. This single-center, retrospective cohort study was conducted at Naresuan University Hospital, Phitsanulok, Thailand, and included patients with end-stage kidney disease (ESKD) receiving maintenance hemodialysis, peritoneal dialysis, or kidney transplantation between June 2022 and June 2023, during the peak of the Omicron variant. Patients who received a single 150/150 mg dose of Tixagevimab-Cilgavimab were compared to those who did not, in terms of time to first COVID-19 infection and hospitalization within 6 months. Cox proportional hazards models were used to evaluate associations, adjusted for age, sex, type 2 diabetes, dyslipidemia, systolic and diastolic blood pressure, serum creatinine, number of COVID-19 vaccine doses, and prior COVID-19 infection. Safety was assessed by comparing creatine kinase (CK) levels before and after treatment using generalized estimating equations (GEE). Of 117 patients, 58 received Tixagevimab-Cilgavimab (mean age 59 ± 15 years); 92% were on dialysis and 8% had undergone kidney transplantation. COVID-19 infection occurred in 10.3% of the LAAB group versus 11.9% in the control group. In the adjusted Cox model, LAAB use was significantly associated with a reduced risk of COVID-19 infection (adjusted HR: 0.20; 95% CI: 0.04–0.95; *p* = 0.043). No variables were significantly associated with hospitalization, although LAAB use showed a non-significant trend toward reduced hospitalization risk (adjusted HR: 0.08; 95% CI: 0.01–1.56; *p* = 0.096). No local or systemic adverse effects were reported. CK levels remained unchanged after administration. Tixagevimab-Cilgavimab was effective in reducing the risk of COVID-19 infection among ESKD patients, without evidence of adverse effects, supporting its use as a prophylactic agent in this high-risk population.

## 1. Introduction

The COVID-19 pandemic has resulted in a substantial number of infections and deaths worldwide, with particularly severe outcomes among patients with end-stage kidney disease (ESKD) receiving kidney replacement therapy (KRT) [1]. Hospitalized COVID-19 patients with ESKD—whether on hemodialysis (HD), peritoneal dialysis (PD), or having undergone kidney transplantation (KT)—and even those with advanced non-dialysis chronic kidney disease (CKD), have demonstrated a markedly higher risk of mortality compared to patients without CKD [2]. The current systematic review and meta-analysis of 22 trials involving 13,910 hemodialysis patients reported a COVID-19 mortality rate of approximately 24% (95% CI: 19–28%), which is higher than that observed in patients not receiving maintenance hemodialysis [3]. ESKD patients have impaired immune function due to uremia, rendering them more susceptible to infections and associated with a higher mortality rate than the general population [4]. Furthermore, there is evidence that these patients, including kidney transplant (KT) recipients receiving immunosuppressive therapy, exhibit a reduced immune response to COVID-19 vaccination [4]. Recent hemodialysis cohort studies have demonstrated high seroconversion rates after two doses of BNT162b2; however, antibody titers and neutralizing activity were significantly lower compared with healthy controls [5,6]. Even when vaccination induces an immune response, it is often weaker and declines more rapidly than in healthy individuals, leading to insufficient protection against infection [1,4]. Consequently, the original COVID-19 vaccines may not provide sustained immunity in patients undergoing renal replacement therapy. To address this gap, long-acting antibodies (LAAB) have been developed to prevent SARS-CoV-2 infection and mitigate disease severity. In clinical trials, Tixagevimab/Cilgavimab for COVID-19 prevention was evaluated in 5197 participants randomized to receive either Tixagevimab/Cilgavimab or placebo. Of these, 3460 participants received Tixagevimab/Cilgavimab and 1737 received placebo. Symptomatic COVID-19 occurred in only 8 of 3441 participants (0.2%) in the Tixagevimab/Cilgavimab group, compared with 17 of 1731 (1.0%) in the placebo group, representing a relative risk reduction of 76.7% (95% CI: 46.0–90.0; *p* < 0.001). At a median follow-up of 6 months, the relative risk reduction increased to 82.8% (95% CI: 65.8–91.4). All 5 cases of severe or critical COVID-19, including 2 deaths, occurred in the placebo group. Adverse events were reported in 35.3% of the Tixagevimab/Cilgavimab group and 34.2% of the placebo group, with most being mild to moderate in severity [7]. The U.S. Food and Drug Administration (FDA) issued an Emergency Use Authorization (EUA) for tixagevimab–cilgavimab (Evusheld) in December 2021 for COVID-19 pre-exposure prophylaxis; however, the EUA was withdrawn in January 2023 due to reduced activity against emerging variants [8]. In Thailand, the Thai FDA approved LAAB for emergency use on 27 June 2022 for adults and adolescents (≥12 years, ≥40 kg) with immunodeficiency [9]. A study in Thailand reported that, among 103 recipients, only 5 (4.9%) developed COVID-19 within six months after injection, all of whom experienced mild disease without hospitalization; reported adverse effects were mild and self-limiting [9]. However, there is currently a lack of robust, real-world evidence evaluating LAAB prophylaxis specifically in ESKD patients receiving KRT, despite their known vulnerability to severe COVID-19 and suboptimal vaccine responses [1,4,9]. Therefore, this study aims to evaluate the effectiveness of tixagevimab–cilgavimab in ESKD patients undergoing KRT compared with non-LAAB controls, focusing on infection rates, disease severity, and safety outcomes. The findings may inform future COVID-19 prevention strategies for this high-risk population.

## 2. Methods

### 2.1. Study Population

This retrospective cohort study was conducted at Naresuan University Hospital, a tertiary referral center in Phitsanulok Province, Thailand. The study population comprised patients with ESKD receiving KRT, including HD, PD, and KT. Participants were divided into two groups: the intervention group consisted of 58 patients who received tixagevimab 150 mg and cilgavimab 150 mg, a combination LAAB therapy, and a historical control group of 59 patients who did not receive LAAB prophylaxis during the same period (non-LAAB). Data were collected between September 2022 and March 2023, during the COVID-19 Omicron variant outbreak in Thailand. All eligible patients during this period who met the inclusion criteria were consecutively enrolled to minimize selection bias, and the same clinical definitions and diagnostic criteria were applied to both groups.

Inclusion criteria were ESRD patients receiving KRT or KT during the outbreak period, aged over 18 years, weighing more than 40 kg, and with no history of COVID-19 infection within 3 months prior to vaccination. Exclusion criteria were metastatic cancer, end-stage liver cirrhosis (Child–Pugh class B or C), pregnancy, and unstable vital signs within 2 weeks prior to vaccination. The sample size was based on the total number of eligible patients available during the study period, as no formal sample size calculation was performed due to the limited population.

### 2.2. Data Collection

The study was approved by the Naresuan University Human Research Ethics Committee (IRB No. P3-0050/2568; approval date: 10 June 2025). Data extracted from the hospital’s electronic medical records included demographic and clinical information (age, sex, comorbidities, cause of ESKD, type of KRT, vital signs), COVID-19 history prior to vaccination, COVID-19 vaccination history, COVID-19 infection within 6 months after vaccination, hospitalization, and symptoms/signs if infected. Laboratory data included complete blood count, blood urea nitrogen, serum creatinine, electrolytes, blood glucose, serum phosphorus, serum albumin, parathyroid hormone, CK, and serum calcium.

Patients were followed for 180 days to evaluate the efficacy of tixagevimab/cilgavimab. The primary outcome was the occurrence of COVID-19 infection. Secondary outcomes were hospitalization due to COVID-19 and adverse events following administration of tixagevimab/cilgavimab. Adverse events were recorded throughout the 6-month follow-up period through clinical assessment and laboratory monitoring.

### 2.3. Statistical Analysis

Baseline characteristics and outcomes were summarized using descriptive statistics. Categorical variables were presented as frequencies and percentages, and continuous variables as mean ± standard deviation (SD) for normally distributed data or median (interquartile range [IQR]) for skewed data. Normality was assessed using skewness and kurtosis. Comparisons between the LAAB and non-LAAB groups were performed using Fisher’s exact test or Pearson’s chi-square test for categorical variables, and Student’s *t*-test or Mann–Whitney U test for continuous variables, as appropriate.

COVID-19 infection and hospitalization rates within 180 days were compared between LAAB and non-LAAB groups using multivariable Cox proportional hazards regression models with risks of COVID-19 infections and hospitalization. Generalized estimating equations were used to compare CK levels at baseline, 3 months, and 6 months between groups. Statistical significance was set at *p* < 0.05. All analyses were conducted using STATA version 18BE (StataCorp LLC, College Station, TX, USA).

## 3. Results

The study included 117 patients with ESKD receiving kidney replacement therapy at Naresuan University Hospital between September 2022 and March 2023 who met the study criteria. Of these, 58 patients received tixagevimab 150 mg plus cilgavimab 150 mg (LAAB group), and 59 patients did not receive LAAB prophylaxis (non-LAAB group). In the LAAB group, 31 patients (53.5%) were on hemodialysis, 23 (39.7%) on peritoneal dialysis, and 4 (6.9%) had undergone kidney transplantation; in the non-LAAB group, 38 (64.4%) were on hemodialysis, 16 (27.1%) on peritoneal dialysis, and 5 (8.5%) had undergone kidney transplantation.

Baseline characteristics are summarized in Table 1. The LAAB group included 33 males (56.9%) and the non-LAAB group 32 males (54.2%) (*p* = 0.853). The mean age was similar between groups (59.7 ± 14.5 vs. 59.1 ± 15.2 years, *p* = 0.831). A history of prior COVID-19 infection was significantly less common in the LAAB group than in the non-LAAB group (15.5% vs. 33.9%, *p* = 0.031). The LAAB group had significantly higher mean systolic and diastolic blood pressures (145.7 ± 26.9 vs. 134.8 ± 18.8 mmHg, *p* = 0.013; and 77.6 ± 17.2 vs. 70.5 ± 15.4 mmHg, *p* = 0.021, respectively). Platelet counts were lower in the LAAB group (219.4 ± 70.5 vs. 250.6 ± 88.2 × 10^9^/L, *p* = 0.037), while other laboratory parameters did not differ significantly between groups.

During follow-up, COVID-19 infection occurred in 6 patients (10.3%) in the LAAB group and 7 patients (11.9%) in the non-LAAB group (*p* = 0.794). Hospitalization for COVID-19 was infrequent, with 1 case (1.7%) in the LAAB group and 4 cases (6.8%) in the non-LAAB group (*p* = 0.364).

**Figure 1:**
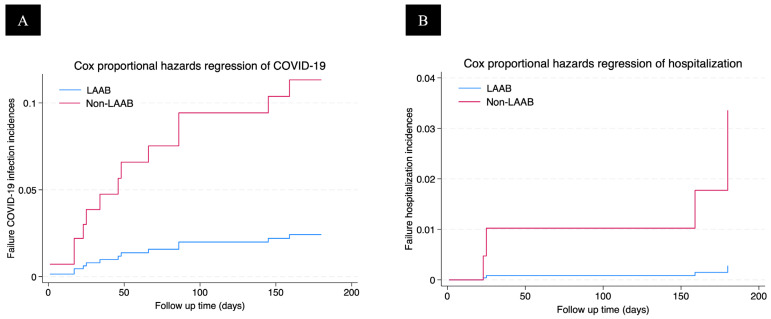
Multivariable Cox proportional hazards regression curves comparing the cumulative incidence of COVID-19 infection (**A**) and COVID-19–related hospitalization (**B**) between patients receiving long-acting antibody (LAAB) prophylaxis and those not receiving LAAB.

In Cox proportional hazards analysis, LAAB administration was associated with a significantly lower risk of COVID-19 infection (adjusted hazard ratio [aHR] 0.20, 95% CI 0.04–0.95, *p* = 0.043) after adjusting for gender, age, type 2 diabetes mellitus, dyslipidemia, history of previous COVID-19 infection, prior COVID-19 vaccine doses, blood pressure, and serum creatinine (Table 2, Figure 1). Hospitalization for COVID-19 showed a non-significant trend toward reduction in the LAAB group (aHR 0.08, 95% CI 0.01–1.56, *p* = 0.096). Two deaths were recorded. One was due to myocardial infarction in the LAAB group, and one was due to severe COVID-19 pneumonia in the non-LAAB group.

**Figure 2:**
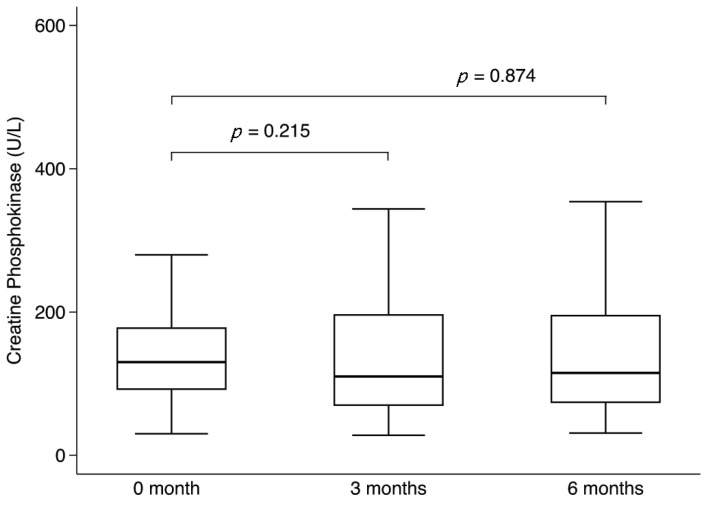
Serum creatine phosphokinase levels at baseline, 3-month, and 6-month follow-up in patients receiving long-acting antibody prophylaxis, analyzed using generalized estimating equations.

Regarding safety outcomes, there were no significant changes in CK levels over the 6-month follow-up. The change from baseline was −29.7 U/L (95% CI −76.6 to 17.2, *p* = 0.215) at 3 months and −4.4 U/L (95% CI −58.5 to 49.7, *p* = 0.874) at 6 months (Figure 2). No local reactions or injection site pain were reported in the LAAB group. No serious adverse effects or deaths were reported in the study.

## 4. Discussion

This study provides real-world evidence that LAAB prophylaxis, when administered in addition to COVID-19 vaccination, was associated with a reduced hazard of COVID-19 infection and a borderline reduction in hospitalization risk among immunocompromised patients with ESKD receiving KRT or KT. These associations remained after adjustment for potential confounders, including sex, age, type 2 diabetes mellitus, dyslipidemia, history of prior COVID-19 infection, number of previous vaccine doses, blood pressure, and serum creatinine. Our findings suggest that, during the Omicron outbreak, LAAB offered an added protective benefit to vaccination in reducing infection risk in this high-risk population. Given their neutralizing activity, LAABs could be considered as an adjunctive preventive strategy during COVID-19 surges or future outbreaks of novel viral pathogens.

ESKD is associated with a substantially higher risk of COVID-19–related mortality compared to patients not receiving maintenance hemodialysis [2,4]. This vulnerability is driven by impaired immunity from uremia and a diminished vaccine response. COVID-19 vaccine responses in dialysis patients vary widely, ranging from 29.6% to 96.4%. Factors such as age, previous COVID-19 infection, use of immunosuppressive therapy, body mass index, and serum albumin levels are associated with vaccine responsiveness in this population [1]. KT recipients face an even greater risk due to chronic immunosuppression, which further reduces vaccine-induced immunity and increases susceptibility to infection compared with patients on HD or PD [10]. In our cohort, the overall COVID-19 infection rate was 11%, lower than the 15–17% reported in prior studies involving similar populations, with and without LAAB use [11]. The proportion of KT recipients receiving LAAB in our center was relatively low, largely due to safety concerns during its initial introduction in Thailand at the time our study was conducted. Tixagevimab–cilgavimab has been developed to supplement vaccine-induced immunity by providing passive neutralizing antibodies. While concerns about safety were raised following its U.S. FDA Emergency Use Authorization [12], subsequent studies have demonstrated efficacy and safety in immunocompromised populations, including those with impaired vaccine responses [9,13,14,15]. Our results add to this body of evidence, supporting its potential role in high-risk ESKD and KT populations.

Tixagevimab and cilgavimab neutralize COVID-19 infection and exhibit an extended half-life due to YTE mutations [16]. In kidney transplant recipients, these agents reduced the rate of COVID-19 infection when used as pre-exposure prophylaxis during the Omicron BA.1/BA.2 phase, with a median half-life of 91 days, but they failed to provide protection against BA.4/5 [17]. In dialysis patients, however, the half-life may extend up to 6 months when a higher dose of tixagevimab/cilgavimab (300 mg/300 mg) is used [14]. Consistent with this, our study showed a failure to prevent COVID-19 infection, with incidence rates of 10.3% in the LAAB group versus 11.9% in the control group receiving the lower 150 mg/150 mg dose over a 6-month follow-up. Because this was a retrospective cohort, we included clinically meaningful risk factors of COVID-19–related outcomes and adjusted for them in multivariable analyses of primary and secondary outcomes. A large observational study of 419 ESKD patients demonstrated that older age and comorbid conditions are independent risks of death in the ESKD population [18]. In addition, a sex difference in COVID-19 symptoms was reported in ESKD patients (25% in men vs. 10% in women) [19]. Furthermore, the number of vaccine doses received yielded a protective effect on COVID-19 mortality in hemodialysis patients [20]. Serum creatinine, which represents muscle mass and nutritional status, was identified as a factor increasing the risk of ventilator support in hemodialysis patients with COVID-19 infection [21,22]. Nonetheless, patients with LAAB prophylaxis experienced a reduced hazard of COVID-19 infection despite vaccination, similar to the findings of Boongird et al., who demonstrated a trend toward delayed infection (median 4.49 [2.81–4.98] vs. 1.96 [1.65–2.91] months; *p* = 0.08) and a significantly reduced hospitalization rate (5.9% vs. 40.0%; *p* = 0.02) [11]. In line with this, we observed a borderline reduction in the hazard of hospitalization (aHR 0.08, *p* = 0.096) and a significant reduction in the hazard of COVID-19 infection (aHR 0.20, *p* = 0.043) in the LAAB group. Importantly, our analysis accounted for history of prior COVID-19 infection and prior vaccine doses, both of which have been shown to influence the risk of COVID-19 infection in previous studies [23]. These findings suggest that LAAB may provide additional protective benefits against COVID-19 infection and hospitalization in the ESKD population during the Omicron era. Before the FDA withdrew its authorization of tixagevimab/cilgavimab due to reduced activity against emerging variants, it had recommended administration every 6 months in immunosuppressed patients [8]. In addition, increasing the dose of tixagevimab/cilgavimab from 150 mg to 600 mg demonstrated a 29% to 74% shift in effectiveness of COVID-19 prevention in immunocompromised patients [24]. The current systematic review and meta-analysis of hemodialysis patients and organ transplant recipients demonstrated the effectiveness and safety of tixagevimab/cilgavimab [15,25].

The safety profile of tixagevimab/cilgavimab in ESKD patients has been well demonstrated, with a low incidence of adverse events [14,26,27]. In our study, no adverse effects were reported among patients receiving LAAB. Previous reports have documented mild events, such as local injection site reactions, pain, fever, or fatigue [14,26]. Importantly, our study showed no significant change in CK levels during the 6-month follow-up. During the early use of LAAB, there was concern regarding potential systemic effects, which justified close monitoring of symptoms and laboratory parameters. However, current evidence suggests that tixagevimab/cilgavimab is not associated with rhabdomyolysis or muscle enzyme elevation. On the contrary, by reducing the risk of COVID-19 infection, LAAB may indirectly lower the risk of rhabdomyolysis, as COVID-19 itself has been associated with muscle injury and rhabdomyolysis [28]. Our study reported two deaths, one related to severe COVID-19 infection in a non-LAAB patient and one from a non–COVID-19–related cause, which seems to be consistent with better outcomes of LAAB neutralizing. Furthermore, in the systematic review and meta-analysis of six studies in ESKD patients using tixagevimab/cilgavimab, lower death than control was yielded with OR: 0.17; 95% CI: 0.03–0.99, *p* = 0.05, I2 = 72% [25].

Given our single-center, retrospective design and modest sample size, the findings should be interpreted with caution and regarded as hypothesis-generating. Generalizability to other regions and care settings may be limited. The sample size (n = 117) constrains statistical power and the precision of effect estimates. To mitigate confounding in this nonrandomized cohort, we adjusted for measured covariates associated with SARS-CoV-2 infection; nevertheless, residual confounding and selection bias remain possible. Optimal prophylaxis conditions—including dose, dosing interval, and timing relative to dialysis sessions—remain uncertain and may vary with circulating variants and patient immune status. Our real-world cohort included both pre-exposure and post-exposure administration. Prospective dose- and schedule-finding studies in ESKD are warranted. In addition, effectiveness against evolving SARS-CoV-2 sublineages (e.g., NB.1.8.1 and related variants) remains uncertain and requires ongoing evaluation. Larger, multicenter prospective studies are needed to define optimal administration conditions and variant-specific effectiveness, including with newer long-acting antibody formulations.

In conclusion, this retrospective cohort study of ESKD patients receiving KRT demonstrated that prophylaxis with tixagevimab–cilgavimab (LAAB) was associated with a reduced hazard of COVID-19 infection and a borderline benefit in reducing hospitalization, without evident adverse outcomes. LAAB may serve as an important adjunctive preventive strategy for ESKD patients at high risk of severe COVID-19, particularly when vaccine-induced immunity is insufficient. Future viral outbreaks may warrant consideration of neutralizing antibodies as an additional preventive measure in this vulnerable population.

## Figures and Tables

**Table 1:** Baseline characteristics.

Characteristics	LAAB	No LAAB	*p*-Value
	(*n* = 58)	(*n* = 59)	
Gender			
Male	33 (56.9)	32 (54.2)	0.853
Female	25 (43.1)	27 (45.8)	
Age (year), Mean ± SD	59.7 ± 14.5	59.1 ± 15.2	0.831
Comorbidity			
Diabetes mellitus	27 (46.6)	34 (57.6)	0.269
Hypertension	58 (100)	55 (93.2)	0.119
Dyslipidemia	48 (82.8)	40 (67.8)	0.086
Cancer	5 (8.6)	2 (3.4)	0.272
History of COVID-19 infection	9 (15.5)	20 (33.9)	0.031
Systolic blood pressure (mmHg), Mean ± SD	145.7 ± 26.9	134.8 ± 18.8	0.013
Diastolic blood pressure (mmHg), Mean ± SD	77.6 ± 17.2	70.5 ± 15.4	0.021
Number of vaccine (doses), Mean ± SD	2.4 ± 0.9	2.2 ± 1.1	0.256
Primary kidney disease			0.277
Diabetes mellitus	21 (36.2)	30 (50.9)	
Hypertension	26 (44.8)	18 (30.5)	
Lupus nephritis	9 (15.5)	8 (13.6)	
Renal stone	1 (1.7)	3 (5.1)	
ADPKD	1 (1.7)	0	
Renal replacement therapy			0.366
Peritoneal dialysis	23 (39.7)	16 (27.1)	
Hemodialysis	31 (53.5)	38 (64.4)	
Kidney transplantation	4 (6.9)	5 (8.5)	
Hospitalization	4 (6.9)	8 (13.6)	0.362
Hemoglobin (g/dL), Mean ± SD	10.5 ± 1.6	10.2 ± 1.7	0.352
White blood cell (10^9^/L), Mean ± SD	73.9 ± 3.6	73.3 ± 1.8	0.910
Platelet (10^9^/L), Mean ± SD	219.4 ± 70.5	250.6 ± 88.2	0.037
BUN (mmol/L), Mean ± SD	41.0 ± 15.5	44.2 ± 22.2	0.374
Serum creatinine (mg/dL), Mean ± SD	8.4 ± 3.6	7.6 ± 3.7	0.273
Serum calcium (mg/dL), Mean ± SD	9.2 ± 1.1	8.9 ± 0.9	0.164
Serum phosphorus (mg/dL), Mean ± SD	4.2 ± 1.5	4.1 ± 1.5	0.669
Serum albumin (g/dL), Mean ± SD	3.6 ± 0.5	3.7 ± 0.7	0.744
iPTH (pg/mL), median (IQR)	215.1 (139.7–528.3)	184.8 (77–356.2)	0.128

Abbreviations: LAAB, Long-acting antibody; ADPKD, Autosomal dominant polycystic kidney disease; BUN, Blood urea nitrogen; iPTH, Intact parathyroid hormone; SD, Standard deviation; IQR, Interquartile range.

**Table 2:** Multivariable Cox proportional hazard regression of COVID-19 infection and hospitalization in the patients who received long-acting antibody.

Potential Factor	COVID-19		Hospitalization	
	Adjusted HR	*p*-Value	Adjusted HR	*p*-Value
Long-acting antibody	0.20 (0.04–0.95)	0.043	0.08 (0.01–1.56)	0.096
Male gender	1.49 (0.40–5.55)	0.550	0.95 (0.12–7.83)	0.963
Age ≥ 60 years	0.56 (0.10–3.01)	0.498	0.78 (0.01–13.34)	0.861
Diabetes mellitus	2.19 (0.42–11.34)	0.351	0.13 (0.01–4.62)	0.264
Dyslipidemia	3.07 (0.61–15.46)	0.175	1.23 (0.14–11.14)	0.853
History of COVID-19 infection	0.12 (0.02–0.91)	0.040	2.72 (0.28–26.62)	0.390
Number of vaccine (doses)	1.39 (0.73–2.63)	0.313	1.06 (0.44–2.51)	0.902
Systolic blood pressure	0.99 (0.96–1.02)	0.620	1.04 (0.97–1.11)	0.287
Diastolic blood pressure	1.08 (1.02–1.13)	0.003	1.06 (0.97–1.16)	0.199
Serum Creatinine	1.18 (0.96–1.45)	0.111	0.92 (0.64–1.32)	0.665

## Data Availability

The datasets generated and analyzed during the current study are available from the corresponding author upon reasonable request.
